# Stroke metrics during the first year of the COVID-19 pandemic, a tale of two comprehensive stroke centers

**DOI:** 10.1038/s41598-023-44277-2

**Published:** 2023-10-11

**Authors:** Lara Carvalho de Oliveira, Ana Ponciano, Nima Kashani, Suzete N. F. Guarda, Michael D. Hill, Eric E. Smith, Jillian M. Stang, Anand Viswanathan, Ashby C. Turner, Aravind Ganesh

**Affiliations:** 1 Department of Neurology, J Philip Kistler Stroke Research Center, Massachusetts General Hospital, Harvard Medical School, Boston, MA USA; 2 Saskatchewan Stroke Program, Department of Neurosurgery, Royal University Hospital, University of Saskatchewan, Saskatoon, SK Canada; 3 Calgary Stroke Program, Department of Clinical Neurosciences, Cumming School of Medicine, University of Calgary, Calgary, AB Canada; 4 Department of Neurology, J Philip Kistler Stroke Research Center, Massachusetts General Hospital, Harvard Medical School, Suite 300, 175 Cambridge Street, Boston, MA 02114 USA

**Keywords:** Epidemiology, Health services, Cerebrovascular disorders, Viral infection

## Abstract

Although a decrease in stroke admissions during the SARS-CoV-2 pandemic has been observed, detailed analyses of the evolution of stroke metrics during the pandemic are lacking. We analyzed changes in stroke presentation, in-hospital systems-of-care, and treatment time metrics at two representative Comprehensive Stroke Centers (CSCs) during the first year of Coronavirus disease 2019 pandemic. From January 2018 to May 2021, data from stroke presentations to two CSCs were obtained. The study duration was split into: period 0 (prepandemic), period 1 (Wave 1), period 2 (Lull), and period 3 (Wave 2). Acute stroke therapies rates and workflow times were compared among pandemic and prepandemic periods. Analyses were adjusted for age, sex, comorbidities, and pre-morbid care needs. There was a significant decrease in monthly hospital presentations of stroke during Wave 1. Both centers reported declines in reperfusion therapies during Wave 1, slowly catching up but never to pre pandemic numbers, and dropping again in Wave 2. Both CSCs experienced in-hospital workflow delays during Waves 1 and 2, and even during the Lull period. Our results highlight the need for proactive strategies to reduce barriers to workflow and hospital avoidance for stroke patients during crisis periods.

## Introduction

In response to the Coronavirus disease 2019 (COVID-19) pandemic, most affected countries implemented varying degrees of social-distancing measures intended to decrease viral transmission and “flatten the curve”^1,2^.

An unintended consequence of social-distancing messaging could be hospital avoidance by patients with medical emergencies, including acute ischemic stroke (AIS), as observed in Hong Kong during the 2009 H1N1 influenza epidemic^3,4^. Social distancing policies may also result in a loss of services and support networks for seniors or patients with disabilities, potentially impairing their ability to seek medical assistance for emergencies^5–7^.

Drops in the number of patients presenting to hospital with AIS or acute coronary syndrome (ACS) occurred^8–11^. A longitudinal retrospective study from 275 stroke centers across 6 continents reported a 7% decline in stroke admissions and 6.1% decline in intravenous thrombolysis, with larger declines observed at high-volume compared to low-volume centers^12^. Stroke patients who did present to emergency departments did so later than they normally would. While not fully understood, this may have been a consequence of delayed appreciation of stroke symptoms while socially isolated or reluctance to go to the hospital out of perceived increased risk of infection^13–15^.

The COVID-19 pandemic introduced a unique set of challenges for running “codes” for AIS and ACS, both at the pre-hospital and in-hospital stages^16^. The risk posed to healthcare providers by the patient’s unknown COVID status—which increases with the prevalence of community transmission^17^ and the known existence of asymptomatic carriers^18^—and the appropriately heightened focus on personal protective equipment (PPE) are perhaps the biggest impediments. Such concerns have led to directives for “protected code strokes”, transmission-prevention protocols to facilitate the safety of both healthcare professionals and patients^19^. An unfortunate and perhaps inevitable consequence of this competing priority was the slowing of workflow processes^20^. In the assessment and management of acute stroke, speed is essential to optimizing patient outcomes^21,22^. Understanding the effects of the recent COVID-19 pandemic on stroke workflow metrics may help us identify areas of potential delays in acute stroke care during a crisis and to better plan future acute stroke care protocols as part of crisis response strategies.

We aimed to investigate changes in stroke hospitalizations, treatment rates, presentation characteristics, and key time metrics for AIS care during the first year of the COVID-19 pandemic across two urban Comprehensive Stroke Centers in Calgary, Canada, and Boston, Massachusetts, United States.

## Methods

### Sources of data

We used linked provincial administrative and clinical registry data capturing stroke-related data on all patients who were seen at the Comprehensive Stroke Centre (CSC) in Calgary, Alberta (Foothills Medical Centre, FMC), and in Massachusetts General Hospital (MGH), in Boston, Massachusetts. For the Canadian data, we primarily relied on administrative registries and electronic health records to analyze data related to stroke patients during the COVID-19 pandemic. Calgary data on all patients hospitalized with AIS during the pre-pandemic and pandemic periods of interest (defined below) were obtained from the Discharge Abstract Database maintained by the provincial health authority (Alberta Health Services Analytics group via the Alberta Strategy for Patient Oriented Research Support Unit) which contains demographic and clinical information on hospital discharges, including comorbidities and need for continuing care like assisted living or nursing home care^23^. MGH data was obtained from the local registry of all AIS patients admitted throughout the study period. The data was derived from Epic (Epic Systems Corporation), an American privately held healthcare software company, which contains all clinical, demographic and discharge information of patients who present to MGH**.** These data are reliable in reporting ischemic stroke and vascular risk factors^24^, with International Classification of Diseases 10 (ICD-10) codes for stroke having accuracies of 92–97%^20^. In this study, we used the following ICD-10 codes to define AIS: G08, H341, I630, I631, I632, I633, I634, I635, I636, I638, I639, I64, and I676 (Supplemental Material, Table 1).

We abstracted patient demographics; stroke severity (National Institutes of Health Stroke Scale score])^25^; pre-hospital workflow (times for stroke onset and hospital arrival, “onset-to-door”); and in-hospital workflow [computed tomography (CT), thrombolysis, endovascular therapy arterial access and reperfusion times]. In Calgary, these metrics were obtained through the Quality Improvement and Clinical Research (QuICR) registry^26,27^, using linked provincial health care numbers for the patients.

### Statistical analyses

The study period was divided into four segments for analysis based on local COVID-19 infection trends: Pre-Pandemic (reference), Wave 1, Lull period, and Wave 2.

Decisions on the cut-offs were made based on changes in local case numbers and community restrictions in each center. In Calgary, decisions on the pre-pandemic period were based on data from the start of the year 2018. Alberta’s first COVID-19 case was identified on 28-February-2020, followed soon by public health restrictions,21 so the (0) “Pre-Pandemic period” was defined as 1-January-2018 to 27-February-2020. We divided the first Pandemic year into three periods based on key changes in terms of reported COVID-19 case counts, timing of public health restrictions, and impact on health services delivery: (1) “Wave 1” (28-February-2020 to 12-May-2020) was characterized by a relatively small number of cases (peak in new daily cases on 29-April-2021 with 315 new cases and a 7-day rolling average of 252 new cases) but with public health restrictions in full force; (2) A relative “Lull” period (13-May-2020 to 20-July-2020) that began with a gradual relaunch strategy and was characterized by a relative flattening of the curve; (3) and “Wave 2” (21-July-2020 to 15-February-2021). Wave 2 was characterized by a major inflection after Thanksgiving gatherings, with new daily cases peaking at 1,887 on 14-Dec-2020, the contact tracing system becoming overwhelmed, and a return of lockdown-style restrictions.

In MGH, the COVID burden started in March, 2020, so the (0) “Pre-Pandemic period” was defined as 2 years prior to 1-March-2020 and the pandemic period was divided into: (1) “Wave 1” (02-March-2020 to 26-May-2020) where we reached 25.000 COVID-19 cases and during when there were school closures, state ‘stay at home’ orders, Boston curfew and masks mandate; (2) “Lull” period (27-May-2020 to 21-October-2020) with phases 2 and 3 of reopening; (3) and “Wave 2” (22-October-2020 to 18-May-2021) with the reimplementation of curfew.

For the primary analyses, we conducted interrupted time-series analyses to compare the total number of patients presenting to emergency departments each month with stroke and the monthly rates of IVT and EVT for stroke (as cases over time) in the Pre-Pandemic and each of the three Pandemic periods. These analyses were adjusted for age, sex, pre-admission continuing care needs, and the presence of any comorbidity. The interrupted time-series analyses were executed using the "itsa" function in STATA^28^. To do so, the data on stroke admissions and treatments were first organized in a time series format for the Calgary and MGH stroke programs, with each row summarizing the stroke admissions, thrombolysis treatments, and endovascular thrombectomies for a single month. Specific time periods of interest for the regression were demarcated using the relevant months as described above. For adjustments, the distribution of admissions each month by age, sex, comorbidities, and continuing care in each month were also included as additional variables.

**Figure 1:**
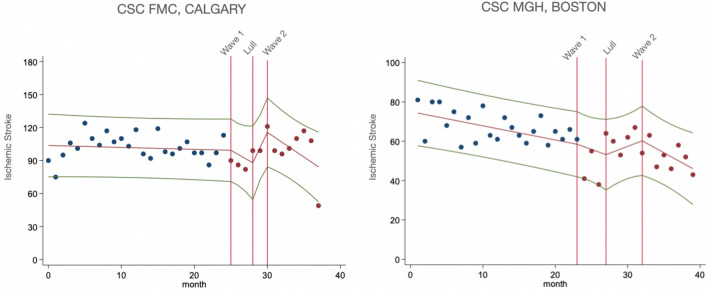
Interrupted time sensitive analyses of stroke admissions from both comprehensive stroke centers. The x-axis represents time, with each month of the study period marked, while the y-axis represents the monthly rate of stroke admissions. Vertical red lines denote the beginnings of the periods of interest, Wave 1, Lull, and Wave 2. In Calgary CSC, monthly hospital presentations of AIS initially decreased during Wave 1, followed by a non-significant increase in the Lull period, and a subsequent drop in Wave 2, though to a lesser extent than during Wave 1. In Boston CSC, AIS monthly hospital admissions steeply decreased during Wave 1, followed by an increase during the Lull period, which did not return to pre-pandemic levels, and a non-significant decline during Wave 2.

**Figure 2:**
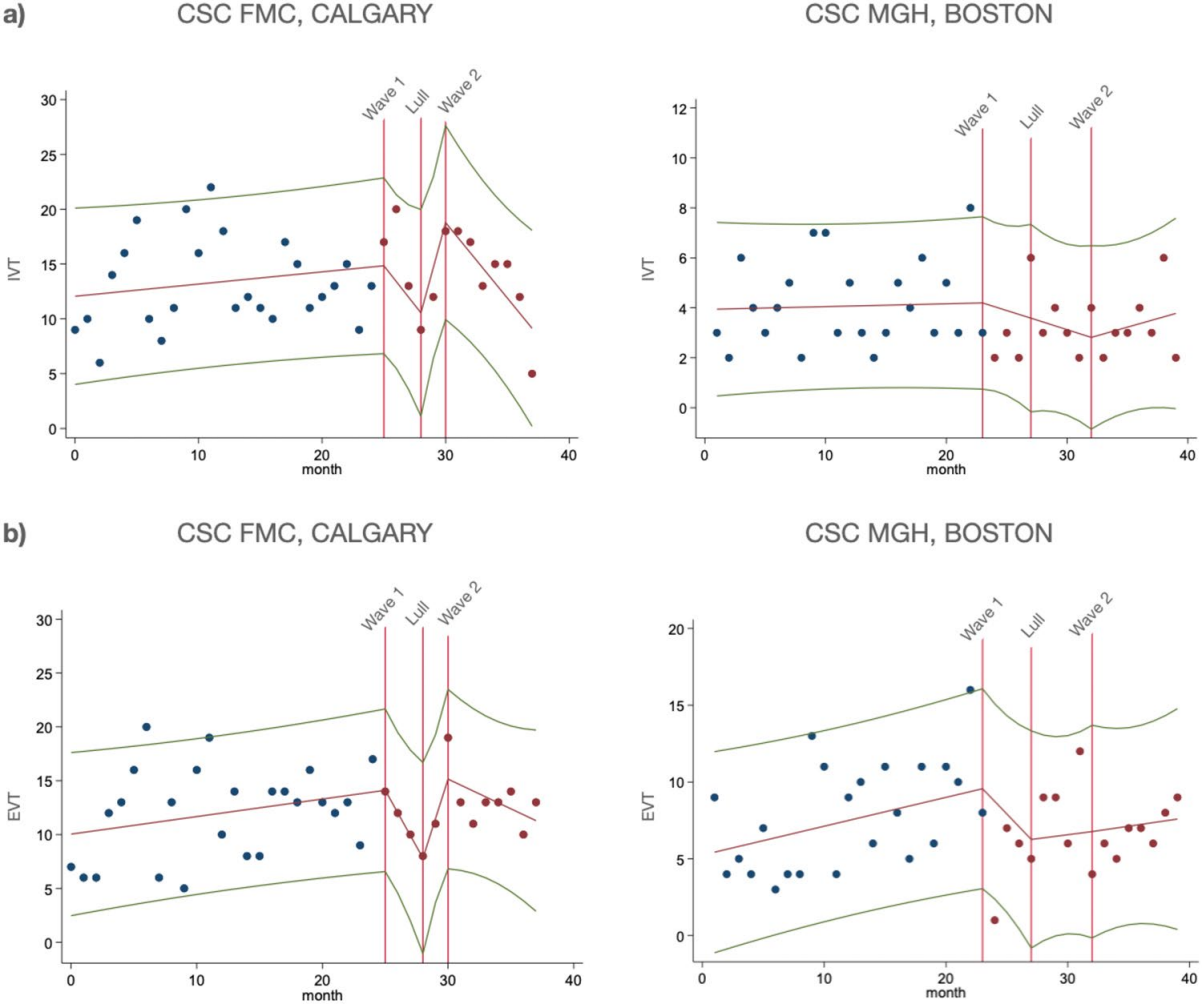
Interrupted time sensitive analyses of (**a**) thrombolysis and (**b**) thrombectomy from both comprehensive stroke centers. The x-axis represents time, with each month of the study period marked, while the y-axis represents the monthly rates of IVT and EVT. Vertical red lines denote the beginnings of the periods of interest, Wave 1, Lull, and Wave 2. In Calgary CSC, there was a non-significant drop in monthly IVT rates during Wave 1, followed by a recovery in the Lull period, and another decline in Wave 2. EVT monthly cases followed a similar trend, first reducing in Wave 1, recovering in the Lull period, and dropping again in Wave 2. In Boston CSC, IVT monthly rates initially dropped in Wave 1 but then rose in Wave 2; and monthly EVT non-significantly decreased in Wave 1, followed by an increase in the Lull period.

For Figs. 1 and 2, we plotted unadjusted data for monthly presentations of ischemic stroke and monthly utilization of thrombolysis and endovascular therapy. Trendlines were generated from interrupted time-series analyses, with knots (potential slope-changes) specified at the start of each time-period of interest. The rationale behind opting for a slope change was based on the nature of the intervention we were investigating, which was the comparison of stroke workflow between the pandemic and pre-pandemic periods. Since the distinctions between each pandemic period were somewhat fluid rather than definitive, we surmised that the impact of the pandemic on stroke workflow was likely to have been gradual, evolving over time as healthcare systems adapted to the changing circumstances. Thus, a slope change allowed us to model the intervention's effect as a gradual shift in the trend of the outcome variables over each study period.

For secondary analyses, we conducted logistic regressions to compare the proportion of all stroke patients who received IVT and EVT in each Pandemic period versus the Pre-Pandemic period. These regression analyses were adjusted for age, sex, pre-admission continuing care needs, and the presence of any comorbidity. We tested for seasonality in each dataset separately for each of the key variables of interest (stroke admissions, IVT, and EVT) using the augmented Dickey–Fuller test to assess whether the variable of interest followed a unit-root process^29,30^. As none of the variables showed seasonality (all p < 0.05), further adjustments for seasonality were not performed. Then, similar logistic regression analyses were performed to compare the proportion of in-hospital mortality and other variables of interest (i.e. age, sex, continuing care needs and comorbidities) in the three Pandemic periods versus the Pre-Pandemic period.

In addition, we compiled any relevant changes in the workflow processes of stroke care that occurred over the first pandemic year at each CSC. We then compared pre- and in-hospital workflow times, in median minutes, and hospital length-of-stay, in median days, using quantile regressions, as linear models were not appropriate due to the non-normality of residuals. These quantile regressions were adjusted for age, sex, continuing care needs, and any comorbidities.

Lastly, we compared the admission NIHSS, in median IQR, in each Pandemic period versus the Pre-Pandemic period using quantile regressions. These quantile regressions were adjusted for age, sex, comorbidities, and continuing care needs.

Overall, these analyses aimed to assess the impact of the Pandemic on stroke-related outcomes, pre-hospital factors, in-hospital mortality, workflow processes, and the severity of stroke cases on admission. Analyses were performed with STATA/MP 16.1 and R Version 4.1.1 (R Foundation for Statistical Computing, Vienna, Austria). Statistical significance was defined as p < 0.05.

### Standard protocol approvals, registrations, data availability and patient consents

All methods were carried out in accordance with relevant guidelines and regulations. The need for informed consent was waived by the University of Calgary Conjoint Health Research Ethics Board (REB20-0769) and by the Partners Human Research Committee Institutional Review Board (IRB00012706). The datasets used and/or analysed during the current study are available from the corresponding author on reasonable request. Additional data are listed in the Supplemental Material. FMC data was approved by the University of Calgary Conjoint Health Research Ethics Board (REB20–0769). In MGH, administrative deidentified data was the primary source of information. This decision allowed us to conduct an observational analysis without directly involving human participants. As a result, the need for evaluation by an ethics committee was waived.

## Results

Baseline characteristics from both centers are described in Table 1. At FMC in Calgary, 3816 patients were analyzed during the study period: 69.31% (n = 2645) patients with ischemic stroke pre-pandemic and 30.69% (n = 1171) during the pandemic period. At MGH in Boston, a total of 2400 patients presented with AIS throughout the study period. From those, 66.12% (n = 1587) were admitted before the pandemic and 33.88% (n = 813) during the pandemic. Importantly, at FMC, 79.9% of all patients who presented with AIS symptoms were hospitalized^31^. The dataset at FMC captures not only patients who required hospitalization at the stroke center due to AIS, but also includes those who were seen at the emergency department but were not hospitalized. On the other hand, in MGH only 1% of patients had the same admit and discharge date, suggesting that the vast majority were either admitted or at least observed overnight. This indicates a high level of hospitalization among AIS patients in the both datasets.

**Table 1:** Baseline characteristics of patients presenting with ischemic stroke before and during the first year of the COVID-19 pandemic in Calgary, CA and in Boston, USA.

Characteristics	Pre-pandemic Jan 1, 2018–Feb 27, 2020	Wave 1 Feb 28, 2020–May 11, 2020	Lull May 12, 2020–July 20, 2020	Wave 2 July 21, 2020–Feb 15, 2021
Comprehensive Stroke Center FMC, Calgary				
AIS, N	2645	207	248	716
Age, median (IQR)	71 (60–81)	73 (63–81)	72 (61–83)	72 (62–82)
Female sex, N (%)	1208 (45.7)	99 (47.8)	122 (49.2)	329 (46.0)
Continuing care needs, N (%)	376 (14.2)	33 (15.9)	32 (12.9)	113 (15.8)
Any comorbidity, N (%)	1,643 (62.1)	125 (60.4)	144 (58.1)	450 (62.9)
Atrial fibrillation, N (%)	371 (16.1)	28 (15.1)	25 (12.0)	70 (11.2)
CAD, N (%)	32 (1.4)	3 (1.6)	2 (1.0)	17 (2.7)
Diabetes mellitus, N (%)	591 (25.7)	46 (24.9)	52 (24.9)	160 (25.6)
Heart failure, N (%)	81 (3.5)	3 (1.6)	5 (2.4)	20 (3.2)
Hypertension, N (%)	1384 (60.2)	104 (56.2)	124 (59.3)	377 (60.2)
Chronic kidney disease, N (%)	35 (1.5)	2 (1.1)	3 (1.4)	14 (2.2)

Characteristics	Pre-pandemic Mar 1, 2018–Mar 1, 2020	Wave 1 Mar 2, 2020–May 26, 2020	Lull May 27, 2020–Oct 21, 2020	Wave 2 Oct 22, 2020–May 18, 2021
Comprehensive Stroke Center MGH, Boston				
AIS, N	1587	141	294	378
Age (mean (SD))	70 (59–80)	69 (58–80)	69.5 (58.25–80)	70 (60–79)
Female sex, N (%)	716 (45.1)	67 (47.5)	135 (45.9)	172 (45.5)
Continuing care needs, N (%)	108 (13.8)	14 (9.9)	19 (6.6)	55 (14.8)
Any comorbidity, N (%)	1255 (79.1)	113 (80.1)	231 (78.6)	299 (79.1)
Atrial fibrillation, N (%)	332 (20.9)	30 (21.3)	63 (21.4)	83 (22.0)
CAD, N (%)	320 (20.2)	28 (19.9)	69 (23.5)	80 (21.2)
Diabetes mellitus, N (%)	471 (29.7)	47 (33.3)	87 (29.6)	109 (28.8)
Heart failure, N (%)	187 (11.8)	13 (9.2)	36 (12.2)	47 (12.4)
Hypertension, N (%)	1059 (66.7)	97 (68.8)	205 (69.7)	267 (70.6)
Chronic kidney disease, N (%)	184 (11.6)	18 (12.8)	55 (18.7)	61 (16.1)

*AIS* acute ischemic stroke, *N* number, *IQR* interquartile range, *SD* standard deviation, *CAD* coronary artery disease.

During the pandemic period, patients presenting to the Calgary CSC were less likely to have atrial fibrillation in Wave 2 [adjusted odds ratio (aOR) 0.61, 95%CI 0.46–0.81] but more likely to have coronary artery disease (aOR 1.99, 95%CI 1.10–3.61); otherwise, there were no significant differences compared to the pre-pandemic period (Supplemental Material, Table 2).

In Boston CSC, patients admitted with AIS during the Lull period were less likely to need continuing care even after adjusting for age and sex (aOR 0.45, 95%CI 0.26–0.74). On the other hand, patients were more likely to have chronic kidney disease during the Lull period (aOR 1.86, 95%CI 1.32–2.59) and Wave 2 (aOR 1.48, 95%CI 1.07–2.03), with no other significant differences in the prevalence of comorbidities compared to the pre-pandemic period (Supplemental Material, Table 3).

In Calgary CSC, monthly hospital presentations of AIS decreased in Wave 1 compared to pre-pandemic period in adjusted analysis for age, sex, comorbidities and continuing care needs (β = −3.14, p < 0.01). Further, there was a non-significant increase in the Lull period and drop in Wave 2, however to a lesser extent than during Wave 1. In Boston CSC, monthly hospital admissions of AIS decreased steeply (β = −6.39, p < 0.001) in Wave 1, then increased in the Lull period (β = 9.20, p < 0.001), although not returning to pre-pandemic levels, and further declined in Wave 2 (β = −3.85, p = 0.04), in adjusted analysis for age, sex and comorbidities. After additionally adjusting for continuing care needs, monthly stroke admissions followed a similar trend during Wave 1 (β = −6.52, p < 0.001) and the Lull (β = 9.12, p < 0.001), however the drop in Wave 2 was no longer significant (β = −3.63, p = 0.07) (Fig. 1).

### Acute treatments or outcomes

Both centers reported similar declines in the incidence of thrombolysis and thrombectomy in Wave 1, slowly catching up in the Lull period, though not to pre-pandemic numbers, and then dropping again in Wave 2. In interrupted time series analysis adjusted for age, sex, comorbidities and continuing care needs, there was a non-significant drop in monthly IVT rates in the Calgary CSC in Wave 1, recovery in the Lull period (β = 4.86, p = 0.02), and another decline in Wave 2 (β = −4.46, p < 0.001). EVT monthly cases followed similar trend, first reducing in Wave 1 (β = −2.59, p < 0.01), recovering in the Lull period (β = 5.23, p < 0.001), and dropping again in Wave 2 (β = −3.68, p = 0.001). In Boston CSC, thrombolysis and thrombectomy presented a similar pattern. IVT monthly rates initially dropped in Wave 1 (β = −0.71, p = 0.04), but then rose in Wave 2 (β = 1.30, p < 0.001); and monthly EVT non-significantly decreased in Wave 1, followed by an increase in the Lull period (β = 2.08, p < 0.05) (Fig. 2).

Among those presenting to the Calgary CSC, IVT was given to 344 (13.0%) of 2645 patients pre-pandemic and to 168 (14.35%) of 1171 patients during the pandemic; and EVT to 313 (11.8%) patients pre-pandemic and to 148 (12.64%) during pandemic. In adjusted analysis, patients presenting during Wave 1 were more likely to receive IVT than in the pre-pandemic period (aOR 1.51, 95%CI 1.04–2.19). In Boston CSC, 99 (6.24%) patients received IVT pre-pandemic and 52 (6.40%) during the pandemic, and 181 (11.41%) and 105 (12.92%) received EVT before and during pandemic, respectively. No differences were observed after adjusting for age, sex, comorbidities, and continuing care needs (Table 2).

**Table 2:** Acute treatments and outcomes of IS patients admitted to hospitals before and during the first year of the COVID-19 pandemic in Calgary, CA and in Boston, USA.

Treatment or outcome	Pre-pandemic Jan 1, 2018–Feb 27, 2020	Wave 1 Feb 28, 2020–May 11, 2020	Lull May 12, 2020–July 20, 2020	Wave 2 July 21, 2020–Feb 15, 2021
Comprehensive Stroke Center FMC, Calgary				
AIS presenting or hospitalized	2645	207	248	716
Thrombolysis, N (%)	344 (13.0)	39 (18.8)	29 (11.7)	100 (14.0)
aOR (95% CI)	Reference	**1.51 (1.04, 2.19)**	0.91 (0.60–1.36)	1.08 (0.85 , 1.38)
Endovascular therapy, N (%)	313 (11.8)	26 (12.6)	33 (13.3)	89 (12.4)
aOR (95% CI)	Reference	1.04 (0.67 , 1.60)	1.17 (0.79 , 1.74)	1.06 (0.82 , 1.37)
Hospital length-of-stay, median days (IQR)	6 (3–14)	6 (3–12)	5 (2–17)	7 (3–19)
Adjusted difference (95% CI)	Reference	0.0 (-1.4 , 1.4)	0.0 (-1.4 , 1.4)	**1.0 (0.2, 1.9)**
*In-hospital mortality (%)	206/2.298 (9.0)	21/185 (11.4)	17/209 (8.1)	63/626 (10.1)
aOR (95% CI)	Reference	1.28 (0.79 , 2.09)	0.90 (0.53 , 1.52)	1.11 (0.82 , 1.50)

Treatment or outcome	Pre-pandemic Mar 1, 2018–Mar 1, 2020	Wave 1 Mar 2, 2020–May 26, 2020	Lull May 27, 2020–Oct 21, 2020	Wave 2 Oct 22, 2020–May 18, 2021
Comprehensive Stroke Center MGH, Boston				
AIS presenting or hospitalized	1587	141	294	378
Thrombolysis, N (%)	99 (6.24)	9 (6.38)	20 (6.8)	23 (6.8)
aOR (95% CI)	Reference	0.92 (0.42 , 1.83)	1.03 (0.59 , 1.73)	0.87 (0.52 , 1.42)
Endovascular therapy, N (%)	181 (11.41)	16 (11.35%)	41 (13.95)	48 (12.7)
aOR (95% CI)	Reference	0.69 (0.38 , 1.18)	0.89 (0.60 , 1.30)	0.83 (0.57 , 1.18)
Hospital length-of-stay, median days (IQR)	4 (2–8)	4 (2–7)	5 (3–8)	5 (3–8)
Adjusted difference (95%CI)	Reference	0.00 (−0.20 , 0.00)	0.00 (0.00 , 0.72)	**1.0 (0.24 , 1.0)**
*In-hospital mortality (%)	143 (9.01)	17 (12.06)	25 (8.5)	31 (8.2)
aOR (95%CI)	Reference	1.46 (0.80 , 2.54)	0.98 (0.59 , 1.59)	0.84 (0.52 , 1.32)

Adjusted for age, sex, any comorbidities and continuing care needs.

*AIS* ischemic stroke, *aOR* adjusted odds ratio**,** *IQR* interquartile range.

*Excluding non-hospitalized minor stroke.

Significant values are in bold.

Median length of stay in-hospital slightly increased in Wave 2 in Calgary CSC (adjusted difference 1.0, 95% CI 0.2–1.9) and in Boston CSC (adjusted difference 1.0, 95%CI 0.24–1.0). Mortality remained stable in both centers (Table 2).

### Time metrics/stroke workflow

**Figure 3:**
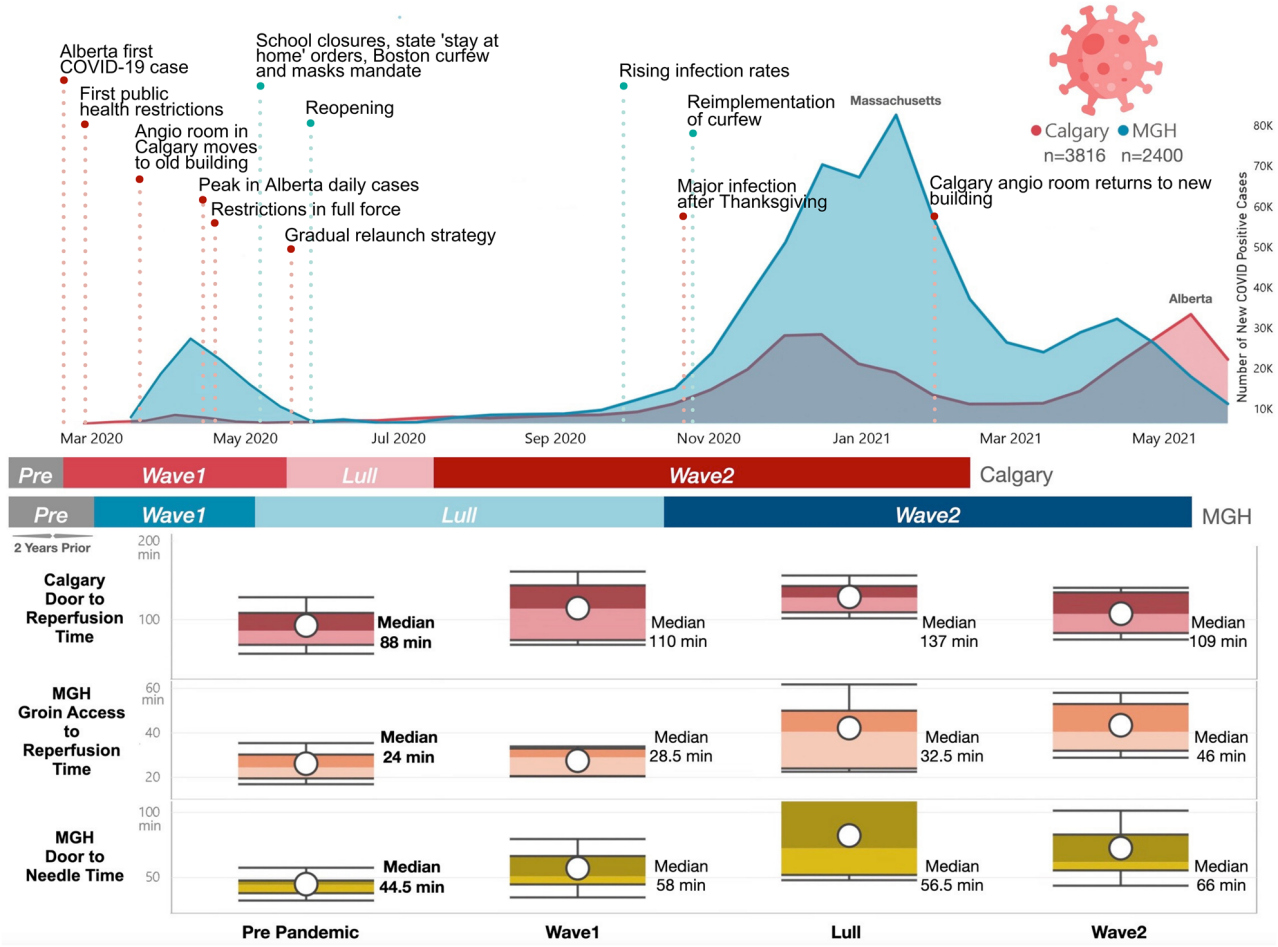
Timeline and metrics of acute ischemic stroke care during the first year of the COVID-19 pandemic in light of infection rates and public policy changes from both CSC, Calgary (in red) and MGH (in blue). The x-axis represents time, tracking the progression of the pandemic along the study period, divided into pre-pandemic period, and Wave 1, Lull and Wave 2. The y-axis represents the number of new COVID cases. Throughout the timeline, notable events such as public policy adjustments in each CSC are indicated, providing context for the observed trends in COVID and stroke care metrics. In the lower part of the figure, we provide details on workflow times for specific acute stroke care metrics that experienced significant changes during the pandemic, such as door-to-reperfusion (in Calgary), and arterial access-to-reperfusion and door-to-reperfusion (in MGH). The workflow times are represented in median (white dots) and IQR (box plots).

During the pandemic, both centers incorporated routine PPE precautions for stroke codes as well as additional cleaning processes. In addition, the Calgary CSC required a geographic change in the location of the angiography suite to meet IP&C precautions for the first part of the study period (Fig. 3). Regarding prehospital response in Calgary CSC, onset-to-door times were prolonged during the pandemic, however this effect was attenuated in adjusted analysis. Moreover, there were no significant differences in in-hospital workflow in door-to-needle times when comparing the 3 pandemic periods with the pre-pandemic period. However, door-to-CT times were a little higher during Wave 1 and Lull period (unadjusted difference 3 min, 95%CI 0.9–5.1). After adjusting for age, sex, comorbidities and continuing care needs, the difference remained significant in the Lull period (adjusted difference 3.7 min, 95%CI 1.3–6.1) (Table 3).

**Table 3:** Pre-hospital and in-hospital workflow of AIS patients who received acute therapies before and during the first year of the COVID-19 pandemic in Calgary, CA.

Characteristics	Pre-pandemic Jan 1, 2018–Feb 27, 2020	Wave 1 Feb 28, 2020–May 11, 2020	Lull May 12, 2020–July 20, 2020	Wave 2 July 21, 2020–Feb 15, 2021
Comprehensive Stroke Center FMC, Calgary				
IVT/EVT recipients, N	498	49	49	146
Pre-hospital response and workflow, median minutes (IQR)				
Onset-to-door time (minutes)	85 (49–181)	88 (51–164)	104 (59–248)	86.5 (55–223)
Adjusted difference, minutes (95%CI)	Reference	−4.8 (−38.9 to 29.3)	15.5 (−18.1 to 49.1)	3.2 (−17.9 to 24.4)
In-hospital workflow, median minutes (IQR)				
Door-to-CT time	17 (13–25)	19.5 (13–31.5)	20 (16–24)	17 (14–22)
Unadjusted difference, minutes (95%CI)	Reference	**3.0 (0.9–5.1)**	**3.0 (0.9–5.1)**	0.0 (−1.3 to 1.3)
Adjusted difference, minutes (95%CI)	Reference	1.8 (−0.7 to 4.2)	**3.7 (1.3–6.1)**	0.2 (−1.3 to 1.7)
Door-to-needle time (IVT)	38 (27–64.5)	38 (26–54)	39 (34–55)	38 (30–52)
Unadjusted difference, minutes (95%CI)	Reference	0.0 (−9.0 to 9.0)	1.0 (−9.1 to 11.1)	0.0 (−6.0 to 6.0)
Adjusted difference, minutes (95%CI)	Reference	0.9 (−8.4 to 10.3)	3.3 (−7.3 to 13.8)	0.2 (−6.0 to 6.4)
CT-to-arterial access time (EVT)	42 (30–59)	40 (32–86)	51 (40–76)	49 (41.5–64)
Unadjusted difference, minutes (95%CI)	Reference	−2.0 (−12.1 to 8.1)	**9.0 (0.1–17.9)**	**7.0 (1.1–12.9)**
Adjusted difference, minutes (95%CI)	Reference	−3.2 (−13.9 to 7.5)	8.7 (−0.7 to 18.1)	**7.3 (1.2–13.5)**
Door-to-arterial access time (EVT)	60 (47–79)	63.4 (47–105)	75 (59–118)	67 (53–92.5)
Unadjusted difference, minutes (95%CI)	Reference	4.0 (−8.2 to 16.2)	**15.0 (4.0–26.0)**	7.0 (−0.2 to 14.2)
Adjusted difference, minutes (95%CI)	Reference	1.1 (−13.1 to 15.2)	**15.3 (2.7–28.0)**	**8.5 (0.1–16.8)**
Arterial access-to-reperfusion time (EVT)	23 (15–35)	29 (22–49)	49 (32–75)	29 (20–52)
Unadjusted difference, minutes (95%CI)	Reference	8.0 (−1.7 to 17.7)	**26.0 (18.4–33.6)**	**6.0 (0.3–11.7)**
Adjusted difference, minutes (95%CI)	Reference	2.4 (−8.2 to 13.0)	**24.4 (16.2–32.6)**	**6.8 (0.6–13.0)**
Door-to-reperfusion time (EVT)	88 (69–125)	110.5 (73–154)	137 (103–181)	109 (83–141)
Unadjusted difference, minutes (95%CI)	Reference	**26.0 (2.1–49.9)**	**49.0 (30.4–67.6)**	**21.0 (6.9–35.1)**
Adjusted difference, minutes (95%CI)	Reference	9.6 (−17.0 to 36.1)	**48.7 (28.2–69.3)**	**17.3 (1.8–32.9)**

Adjusted for age, sex, any comorbidities and continuing care needs.

*N* number, *IVT* intravenous thrombolysis, *EVT* endovascular therapy, *IQR* interquartile range, *CT* computed tomography, *CI* confidence interval.

Significant values are in bold.

Compared to pre-pandemic, median times for CT-to-arterial access were prolonged during Lull period (unadjusted difference 9 min, 95%CI 0.1–17.9) and Wave 2 (unadjusted difference 7 min, 95%CI 1.1–12.9), and remained significantly higher in Wave 2 in the adjusted analysis (adjusted difference 7.3 min, 95%CI 1.2–13.5) (Table 3).

Median times for EVT workflow also rose during Wave 2, when door-to-arterial access, arterial access-to-reperfusion and door-to-reperfusion were 8.5 (95%CI 0.1–16.8), 6.8 (95%CI 0.6–13) and 17.3 min (95%CI 1.8–32.9) slower, respectively. The Lull period experienced a similar trend with differences in the median times for door-to-arterial access, arterial access-to-reperfusion and door-to-reperfusion of 15.3 (95%CI 2.7–28.0), 24.4 (95%CI 16.2–32.6) and 48.7 min (95%CI 28.2–69.3), respectively (Table 3).

Time metrics reported in Boston CSC also faced significant changes during the pandemic periods compared to pre-pandemic. Although prehospital response (onset-to-door time) did not significantly change, in-hospital workflow experienced some delays. During Wave 1 there were significantly prolonged times for thrombolysis and thrombectomy workflows, such as door-to-needle (adjusted difference 16 min, 95%CI 1.0–34.3) and CT-to-arterial access times (adjusted difference 8.9 min, 95%CI 3.2–16.1) (Table 4).

**Table 4:** Pre-hospital and in-hospital workflow of AIS patients who received acute therapies before and during the first year of the COVID-19 pandemic in Boston, USA.

Characteristics	Pre-pandemic Mar 1, 2018–Mar 1, 2020	Wave 1 Mar 2, 2020–May 26, 2020	Lull May 27, 2020–Oct 21, 2020	Wave 2 Oct 22, 2020–May 18, 2021
Comprehensive Stroke Center MGH, Boston				
IVT/EVT recipients, N	280	25	61	71
Pre-hospital response and workflow, median minutes (IQR)				
Onset-to-door time (minutes)	255 (91–574)	268 (122.8–674.2)	243 (123.5–569.2)	220 (80–467.5)
Adjusted difference, minutes (95%CI)	Reference	34.6 (−31.4 to 116.8)	−3.9 (−36.1 to 37.8)	−13.2 (−50.5 to 11.4)
In-hospital workflow, median minutes (IQR)				
Door-to-CT time	71 (28–188)	66 (33.3–142.8)	100 (39–185)	70 (34–228)
Unadjusted difference, minutes (95%CI)	Reference	−4 (−23.8 to 22.6)	29 (−6.1 to 49.4)	−1 (−20.8 tob35.6)
Adjusted difference, minutes (95%CI)	Reference	−6.4 (−21.8 to 9.3)	**24.3 (3.4–43)**	3.5 (−16.7 to 23.2)
Door-to-needle time (IVT)	44.5 (32.3–62.3)	58 (43–89)	56.5 (49.5–121.2)	66 (45–102)
Unadjusted difference, minutes (95%CI)	Reference	13 (−1.1 to 46.8)	**11 (9.9–49.0)**	**21 (9.5–35.5)**
Adjusted difference, minutes (95%CI)	Reference	**16 (1.0–34.3)**	12.3 (−0.3 to 49.7)	**13 (1.9–36.4)**
CT-to-arterial access time (EVT)	63 (44–81)	67 (62–87)	47.5 (35.8–74)	73 (60.5–85)
Unadjusted difference, minutes (95%CI)	Reference	4 (−8.5 to 25.2)	−11 (−30.3 to 6.4)	8 (−5.0 to 17.3)
Adjusted difference, minutes (95%CI)	Reference	**8.9 (3.2–16.1)**	−7.1 (−26.7 to 12.6)	11.3 (−0.6 to 26)
Door-to-arterial access time (EVT)	63 (26–97)	64.5 (33.8–132.5)	55 (13–110)	69 (15–96.3)
Unadjusted difference, minutes (95%CI)	Reference	16 (−29.7 to 67.7)	−8 (−39.3 to 15.3)	2 (−26.8 to 19.9)
Adjusted difference, minutes (95%CI)	Reference	17.7 (−29.1 to 77.2)	−8.20 (−37.8 to 20.4)	11.9 (−20.9 to 31.9)
Arterial access-to-reperfusion time (EVT)	24 (16–43.5)	28.5 (22.3–33)	32.5 (18.8–50)	46 (28.5–56)
Unadjusted difference, minutes (95%CI)	Reference	4 (−0.9 to 10.5)	**8 (0.3–19.7)**	**22 (9.5–29.5)**
Adjusted difference, minutes (95%CI)	Reference	6.5 (−0.6 to 12.2)	**11.9 (3.5–17.6)**	**24.1 (12.9–30.9)**
Door-to-reperfusion time (EVT)	96 (54–164)	73 (56.3–141.5)	91 (47–167.5)	106 (63.8–139)
Unadjusted difference, minutes (95%CI)	Reference	−18 (−36.9 to 27.9)	−4 (−13.7 tob36.7)	10 (−7.7 to 41.3)
Adjusted difference, minutes (95%CI)	Reference	−17 (−34.6 to 23.7)	4.5 (−19.7 to 46.4)	12.6 (−8.0 to 36.4)

Adjusted for age, sex, any comorbidities and continuing care needs.

*N* number, *IVT* intravenous thrombolysis, *EVT* endovascular therapy, *IQR* interquartile range, *CT* computed tomography, *CI* confidence interval.

Significant values are in bold.

The CSC in Boston also experienced delays in the Lull period and Wave 2 compared to the pre-pandemic period. Door-to-CT time during the Lull period increased (adjusted difference 24.3 min, 95%CI 3.4–43). Door-to-needle times were also prolonged in the Lull period in the unadjusted analysis in 11 min (95% CI 9.9–49) and Wave 2 in the adjusted analysis in 13 min (95% CI 1.9–36.4) (Table 4).

Moreover, EVT workflow in Boston CSC such as arterial access-to-reperfusion times were also prolonged in the Lull period in 11.9 min (95%CI 3.5–17.6). Significant delays occurred during Wave 2, when arterial access-to-reperfusion times significantly increased (adjusted difference 24.1 min, 95%CI 12.9–30.99) (Table 4).

### Stroke severity

Baseline NIHSS remained stable with no overall differences were found between pandemic and pre-pandemic, except in Wave 1. During this period in Calgary CSC, all patients treated with acute therapies and also EVT only recipients had significantly lower NIHSS scores, of 4.5 and 6.1 points, in the adjusted analyses, respectively. As for the CSC in Boston, only patients treated with IVT had significantly lower NIHSS (adjusted difference −4.64 points, 95%CI −8.82, −1.69) (Supplemental Material, Table 4).

## Discussion

In two different stroke comprehensive centers in two countries during the first year of the COVID-19 pandemic, there was a similar pattern of decreasing admissions and prolonged workflow times during the beginning of the pandemic then modestly improving during the Lull period, but never to pre-pandemic numbers. The steep decrease may be related to lockdown measures and fear of presenting to emergency departments. Other articles reporting decreases in AIS presentations have generally studied presentation trends or have focused on in-hospital stroke treatment rates^31–33^, but our study adds to the literature by linking institutional stroke workflow changes with a parallel comparison of high-quality complete data from two leading CSCs in different countries.

Our research corroborates previous studies^31,33^ on the evolution of acute stroke care during the COVID-19 pandemic. However, it also offers a unique perspective by analyzing two large CSCs located in different countries, yet exhibiting similar trends.

In the context of the existing literature, our study aligns with prior research indicating a decline in the volume of stroke hospitalizations and IVT during the initial phase of the COVID-19 pandemic^33^. Additionally, Nogueira et al. observed a recovery in stroke hospitalizations, but not IVT volume, in the later phase of the pandemic, similar to our findings at MGH CSC^33^.

Going further, our results can also be compared to previous epidemics, and we can identify several potential reasons for the observed delays during the COVID-19 pandemic. Notably, Lau et al. reported that in the H1N1 outbreak a great proportion of the population demonstrated avoidance behaviours^3^, and Chang et al. suggested that during the SARS epidemic the fear of going to the hospital due to the risk of infection significantly affected accessibility to quality care^8^. Moreover, public health measures including quarantine may have contributed to the increase in the levels of concern among citizens curing the COVID-19 pandemic. These factors collectively may have contributed to the impact on our CSCs, leading to the changes noted in stroke metrics.

It is also worth considering the influence of safety measures that were implemented during the pandemic, such as the protected code stroke and the use of PPE. These strategies aimed to ensure safety not only for patients, but also for healthcare workers^19^, resulting in structural changes within the CSCs that could further influence stroke metrics.

A main aspect of this study to note is the similarity between cohorts despite different geographic location, as well as the similarity of workflow patterns. These results raise concern for a broader decline in stroke presentations and treatment trends. The decline in the rate of patients presenting with AIS also led to delays in workflow times and prolonged length of stay. However, neither CSC experienced significantly higher odds of in-hospital mortality. We also showed that this decline continued to a lesser extent after the early COVID-19 pandemic, which could reflect greater hospital avoidance early on rather than a true decline in stroke rates. Further support for this hospital avoidance hypothesis came from a recent population study in Alberta, Canada which “found” some of the missing strokes during the pandemic in the form of increased out-of-hospital stroke deaths^31^.

The observed delays in stroke workflow times were likely impacted by in-hospital procedural changes in response to the pandemic. While the Canadian center noticed geographic change in the angiography room, in Boston CSC most adjustments were related to sanitation procedures in care settings, which could have contributed to the changes observed.

This article has some limitations. First, workflow data are only available from patients who received acute therapy, which may underestimate resulting times. Also, we do not have information on individual patients’ COVID infection status which may also influence stroke metrics and severity. In addition, the observed workflow delays may also relate to additional challenges in information gathering and treatment decision-making that occurred during the pandemic, which cannot to be completely captured in this type of analysis^32^.

Moreover, in our study, we did not observe any significant changes in in-hospital mortality rates among stroke patients. However, an individual patient meta-analysis, which included data from 83 published and unpublished studies, along with authors' data from the first pandemic wave, reported an increase in in-hospital stroke mortality^31^. Their recruitment period ranged from February to July 2020^34^. Similar results were reported in another systematic review and meta-analysis that enrolled patients from December 2019 to September 2020^35^. We hypothesize that the decrease of in-hospital mortality might be at least partly attributed to an increase of out of hospital mortality. Supporting this, Ganesh et al. reported that the proportion of all stroke deaths in Alberta that occurred out of hospital increased significantly during the first year of the pandemic except from July 21, 2020 to Oct. 11, 2020^31^. In light of the conflicting results and the ongoing nature of the pandemic, we acknowledge the need for further research to delve deeper into the reasons behind the varying mortality outcomes observed in different studies.

In conclusion, both comprehensive stroke centers had fewer stroke presentations and acute treatments administered, with an increase of length of stay during the study period. However, no changes of in-hospital mortality were observed. Strategies must be taken to continue to motivate patients to seek care even during pandemic and stroke protocols should accommodate measures to mitigate inefficiencies.

### Supplementary Information

Supplementary Table 1.

Supplementary Table 2.

Supplementary Table 3.

Supplementary Table 4.

## Data Availability

All data used for this study have been stored in a deidentified database, and the programming code is stored. Requests for access to the data or code, when accompanied by a proposal of appropriate rigor, will be considered by the corresponding author and pending approval from the local REB and IRB.
