# Supplementary material for: Stroke metrics during the first year of the COVID-19 pandemic, a tale of two comprehensive stroke centers
Source: Sci Rep. 2023 Oct 11;13:17171. doi: 10.1038/s41598-023-44277-2 (PMC10567785; doi:10.1038/s41598-023-44277-2)
Supplement: Supplementary file 1 — Supplementary Table 1. [file 41598_2023_44277_MOESM1_ESM.docx]

**Supplementary Table 1. International Classification of Diseases 10 (ICD-10) diagnostic codes used for identifying cases**

| **ICD-10** | **Description** |
| --- | --- |
| **G08** | Intracranial and Intraspinal Phlebitis and Thrombophlebitis |
| **H341** | Central Retinal Artery Occlusion |
| **I630** | Cerebral Infarction Due To Thrombosis Of Precerebral Arteries |
| **I631** | Cerebral Infarction Due To Embolism Of Precerebral Arteries |
| **I632** | Cerebral Infarction Due To Unspecified Occlusion Or Stenosis Of Precerebral Arteries |
| **I633** | Cerebral Infarction Due To Thrombosis Of Cerebral Arteries |
| **I634** | Cerebral Infarction Due To Embolism Of Cerebral Arteries |
| **I635** | Cerebral Infarction Due To Unspecified Occlusion Or Stenosis Of Cerebral Arteries |
| **I636** | Cerebral Infarction Due to Cerebral Venous Thrombosis, Nonpyogenic |
| **I638** | Other Cerebral Infarction |
| **I639** | Cerebral Infarction, Unspecified |
| **I64*** | *** Stroke, Not Specified As Haemorrhage Or Infarction (see note below) |
| **I676** | Nonpyogenic Thrombosis Of Intracranial Venous System |

*** NOTE: The decision to place I64 under the Ischemic group is based on the following rationale: Coding guidelines direct health information management systems to assume “ischemic” if hemorrhagic stroke has been ruled out (there is no supporting documentation of a hemorrhagic stroke). Hemorrhagic strokes are fairly well documented. From the Canadian Institutes of Health Information (CIHI) eQuery 44220: One valid circumstance [for coding I64] is when diagnostic imaging has not yet been performed and another is when any transfer information does not indicate the type of stroke. For the majority of cases, the coder can locate the information required to assign a code for hemorrhagic stroke or ischemic stroke from the documentation. Despite specific resources (CIHI eLearning Different Codes for Different Strokes, eQueries), I64 is still frequently assigned. In such cases, the stroke was most likely ischemic.
