# Supplementary material for: Stroke metrics during the first year of the COVID-19 pandemic, a tale of two comprehensive stroke centers
Source: Sci Rep. 2023 Oct 11;13:17171. doi: 10.1038/s41598-023-44277-2 (PMC10567785; doi:10.1038/s41598-023-44277-2)
Supplement: Supplementary file 2 — Supplementary Table 2. [file 41598_2023_44277_MOESM2_ESM.docx]

**Supplementary Table 2. Adjusted and unadjusted baseline variables of patients presenting with ischemic stroke before and during the first year of the COVID-19 pandemic in Calgary, CA**

| **Characteristics** | **Prepandemic**  Jan 1, 2018 -  Feb 27, 2020 | **Wave 1**  Feb 28, 2020 -  May 11, 2020 | **Lull**  May 12, 2020 -  July 20, 2020 | **Wave 2**  July 21, 2020 -  Feb 15, 2021 |
| --- | --- | --- | --- | --- |
| **Comprehensive Stroke Center FMC, CALGARY** | | | | |
| **Age, years** | | | | |
| Unadjusted difference (95% CI) | Ref. | -0.96 (-3.12-1.23) | -1.16 (-3.16-0.84) | -1.09 (-2.36-0.18) |
| **Sex, female** | | | | |
| Unadjusted OR (95% CI) | Ref. | 0.92 (0.69-1.22) | 0.87 (0.67-1.13) | 0.99 (0.84-1.17) |
| Age-adjusted OR (95% CI) | Ref. | 0.93 (0.70-1.24) | 0.88 (0.68-1.15) | 1.01 (0.85-1.19) |
| **Continuing Care Needs** | | | | |
| Unadjusted OR (95% CI) | Ref. | 1.14 (0.78-1.69) | 0.89 (0.61-1.32) | 1.13 (0.90-1.42) |
| *Adjusted OR (95% CI) | Ref. | 1.07 (0.70-1.64) | 0.74 (0.48-1.13) | 1.05 (0.82-1.35) |
| **Atrial Fibrillation** | | | | |
| Unadjusted OR (95% CI) | Ref. | 0.93 (0.61-1.41) | 0.71 (0.46-1.09) | **0.65 (0.50-0.86)** |
| *****Adjusted OR (95% CI) | Ref. | 0.89 (0.58-1.36) | 0.66 (0.42-1.03) | **0.61 (0.46-0.81)** |
| **Coronary Artery Disease** | | | | |
| Unadjusted OR (95% CI) | Ref. | 1.17 (0.35-3.85) | 0.68 (0.16-2.88) | **1.98 (1.09-3.58)** |
| *****Adjusted OR (95% CI) | Ref. | 1.18 (0.36-3.88) | 0.69 (0.16-2.91) | **1.99 (1.10-3.61)** |
| **Chronic Kidney Disease** | | | | |
| Unadjusted OR (95% CI) | Ref. | 0.71 (0.17-2.96) | 0.94 (0.29-3.09) | 1.48 (0.79-2.77) |
| *****Adjusted OR (95% CI) | Ref. | 0.71 (0.17-2.96) | 0.95 (0.29-3.10) | 1.48 (0.79-2.76) |
| **Diabetes Mellitus** | | | | |
| Unadjusted OR (95% CI) | Ref. | 0.96 (0.68-1.35) | 0.96 (0.69-1.33) | 0.99 (0.81-1.21) |
| *Adjusted OR (95% CI) | Ref. | 0.95 (0.67-1.35) | 0.96 (0.69-1.33) | 0.98 (0.80-1.21) |
| **Heart Failure** | | | | |
| Unadjusted OR (95% CI) | Ref. | 0.45 (0.14-1.44) | 0.67 (0.27-1.67) | 0.90 (0.55-1.49) |
| *Adjusted OR (95% CI) | Ref. | 0.45 (0.14-1.45) | 0.67 (0.27-1.68) | 0.80 (0.55-1.49) |
| **Hypertension** | | | | |
| Unadjusted OR (95% CI) | Ref. | 0.85 (0.63-1.15) | 0.96 (0.72-1.29) | 1.00 (0.83-1.20) |
| *Adjusted OR (95% CI) | Ref. | 0.81 (0.59-1.11) | 0.93 (0.69-1.26) | 0.96 (0.79-1.16) |
| **Any Comorbidity** | | | | |
| Unadjusted OR (95% CI) | Ref. | 0.93 (0.70-1.24) | 0.84 (0.65-1.10) | 1.03 (0.87-1.22) |
| *Adjusted OR (95% CI) | Ref. | 0.89 (0.66-1.20) | 0.80 (0.61-1.06) | 0.99 (0.83-1.18) |

OR: odds ratio
*Adjusted for age and sex
