# Supplementary material for: Stroke metrics during the first year of the COVID-19 pandemic, a tale of two comprehensive stroke centers
Source: Sci Rep. 2023 Oct 11;13:17171. doi: 10.1038/s41598-023-44277-2 (PMC10567785; doi:10.1038/s41598-023-44277-2)
Supplement: Supplementary file 3 — Supplementary Table 3. [file 41598_2023_44277_MOESM3_ESM.docx]

**Supplementary Table 3. Adjusted and unadjusted baseline variables of patients presenting with ischemic stroke before and during the first year of the COVID-19 pandemic in Boston, USA**

| **Characteristics** | **Prepandemic**  Jan 1,2018 -  Mar 1,2020 | **Wave 1**  Mar 2, 2020 -  May 26,2020 | **Lull**  May 27, 2020 -  Oct 21,2020 | **Wave 2**  Oct 22,2020 -  May 18,2021 |
| --- | --- | --- | --- | --- |
| **Comprehensive Stroke Center MGH, BOSTON** | | | | |
| **Age, years** | | | | |
| Unadjusted difference (95% CI) | Ref. | -1 (-7.86 – 5.86) | 0 (-12.96 – 5.46) | 0 (-3.38 – 9.88) |
| **Sex, female** | | | | |
| Unadjusted OR (95% CI) | Ref. | 1.10 (0.78 – 1.55) | 1.03 (0.80 – 1.33) | 1.02 (0.81 – 1.27) |
| Age-adjusted OR (95% CI) | Ref. | 1.12 (0.79 – 1.59) | 1.05 (0.82 – 1.36) | 1.02 (0.81 – 1.28) |
| **Continuing Care Needs** | | | | |
| Unadjusted OR (95% CI) | Ref. | 0.69 (0.37 – 1.20) | **0.44 (0.26 – 0.71)** | 1.08 (0.76 – 1.53) |
| *Adjusted OR (95% CI) | Ref. | 0.70 (0.37 – 1.24) | **0.45 (0.26 – 0.74)** | 1.12 (0.78 – 1.61) |
| **Atrial Fibrillation** | | | | |
| Unadjusted OR (95% CI) | Ref. | 1.02 (0.66 – 1.54) | 1.03 (0.76 – 1.39) | 1.06 (0.81 – 1.39) |
| *****Adjusted OR (95% CI) | Ref. | 1.05 (0.67 – 1.61) | 1.09 (0.79 – 1.48) | 1.07 (0.80 – 1.41) |
| **Coronary Artery Disease** | | | | |
| Unadjusted OR (95% CI) | Ref. | 0.98 (0.63 – 1.49) | 1.21 (0.90 – 1.63) | 1.06 (0.80 – 1.39) |
| *****Adjusted OR (95% CI) | Ref. | 1.01 (0.64 – 1.55) | 1.28 (0.94 – 1.73) | 1.07 (0.80 – 1.41) |
| **Chronic Kidney Disease** | | | | |
| Unadjusted OR (95% CI) | Ref. | 1.12 (0.64 – 1.83) | **1.75 (1.25 – 2.43)** | **1.47 (1.07 – 2.00)** |
| *****Adjusted OR (95% CI) | Ref. | 1.14 (0.65 – 1.87) | **1.86 (1.32 – 2.59)** | **1.48 (1.07 – 2.03)** |
| **Diabetes Mellitus** | | | | |
| Unadjusted OR (95% CI) | Ref. | 1.18 (0.82 – 1.70) | 1.00 (0.76 – 1.30) | 0.96 (0.75 – 1.23) |
| *Adjusted OR (95% CI) | Ref. | 1.19 (0.82 – 1.71) | 1.00 (0.76 – 1.31) | 0.96 (0.75 – 1.23) |
| **Heart Failure** | | | | |
| Unadjusted OR (95% CI) | Ref. | 0.76 (0.40 – 1.32) | 1.04 (0.70 – 1.51) | 1.06 (0.75 – 1.48) |
| *Adjusted OR (95% CI) | Ref. | 0.76 (0.40 – 1.34) | 1.09 (0.73 – 1.59) | 1.06 (0.74 – 1.50) |
| **Hypertension** | | | | |
| Unadjusted OR (95% CI) | Ref. | 1.10 (0.76 – 1.61) | 1.15 (0.88 – 1.51) | 1.20 (0.94 – 1.54) |
| *Adjusted OR (95% CI) | Ref. | 1.14 (0.78 – 1.69) | 1.23 (0.93 – 1.63) | 1.22 (0.95 – 1.59) |
| **Any Comorbidity** | | | | |
| Unadjusted OR (95% CI) | Ref. | 1.07 (0.70 – 1.67) | 0.97 (0.72 – 1.32) | 1.00 (0.76 – 1.33) |
| *Adjusted OR (95% CI) | Ref. | 1.12 (0.71 – 1.81) | 1.04 (0.75 – 1.45) | 1.00 (0.75 – 1.36) |

OR: odds ratio
*Adjusted for age and sex
