# Supplementary material for: Stroke metrics during the first year of the COVID-19 pandemic, a tale of two comprehensive stroke centers
Source: Sci Rep. 2023 Oct 11;13:17171. doi: 10.1038/s41598-023-44277-2 (PMC10567785; doi:10.1038/s41598-023-44277-2)
Supplement: Supplementary file 4 — Supplementary Table 4. [file 41598_2023_44277_MOESM4_ESM.docx]

**Supplementary Table 4. Stroke severity of patients who received acute therapies before and during the first year of the COVID-19 pandemic, in Calgary, CA and in Boston, USA**

| **Stroke severity, median (IQR)** | **Prepandemic**  Jan 1, 2018 -  Feb 27, 2020 | **Wave 1**  Feb 28, 2020 -  May 11, 2020 | **Lull**  May 12, 2020 -  July 20, 2020 | **Wave 2**  July 21, 2020 -  Feb 15, 2021 |
| --- | --- | --- | --- | --- |
| **CSC CALGARY** | | | | |
| **Pre-treatment NIHSS (all)** | 12 (6-19) | 8 (6-14) | 13 (7-17) | 11.5 (6-18) |
| Adjusted difference, points (95%CI) | Reference | **-4.5 (-8.0 , -0.9)** | 1.2 (-2.4 , 4.8) | -1.4 (-3.6 , 0.9) |
| **Thrombolysis recipients** | 11 (6-18) | 8 (6-14) | 12 (7-14) | 10 (5-17) |
| Adjusted difference, points (95%CI) | Reference | -1.0 (-5.0 , 3.0) | 2.0 (-2.5 , 6.5) | 0.0 (-2.6 , 2.6) |
| **EVT recipients** | 16 (10-20) | 10.5 (6.5-15.5) | 14 (8-17) | 15 (9-20) |
| Adjusted difference, points (95%CI) | Reference | **-6.1 (-10.3 , -1.9)** | -1.6 (-5.3 , 2.1) | -1.1 (-3.6 , 1.3) |
| **Stroke severity, median (IQR)** | **Prepandemic**  Mar 1,2018 -  Mar 1,2020 | **Wave 1**  Mar 2, 2020 -  May 26,2020 | **Lull**  May 27, 2020 -  Oct 21,2020 | **Wave 2**  Oct 22,2020 -  May 18,2021 |
| **CSC MGH, BOSTON** | | | | |
| **Pre-treatment NIHSS (all)** | 4 (1-12) | 4 (2-10) | 4 (1-12) | 4 (1-12) |
| Adjusted difference, points (95%CI) | Reference | -0.20 (-1.25 , 0.71) | 0.13 (-0.79 , 1.11) | 0.40 (-0.84 , 0.98) |
| **Thrombolysis recipients** | 12 (6-18) | 5 (4-8) | 11 (8-13) | 14 (9-20) |
| Adjusted difference, points (95%CI) | Reference | **-4.64 (-8.82 , -1.69)** | 0.46 (-4.16 , 2.94) | 1.4 (-3.41 , 6.51) |
| **EVT recipients** | 17 (11-21) | 15 (10-24) | 15 (10-21) | 17 (10-20) |
| Adjusted difference, points (95%CI) | Reference | -1.06 (-8.15 , 6.91) | -2.32 (5.20 , 3.00) | -0.25 (-2.67 , 1.95) |

IQR: interquartile range; NIHSS: National Institutes of Health Stroke Scale; CI: confidence interval EVT: endovascular therapy; aOR: adjusted odds ratio

Adjusted for age, sex, any comorbidities and continuing care needs
